# Recombinant TadZ from the type IVc pilus system induces protective immunity against virulent *Aeromonas hydrophila* in channel catfish (*Ictalurus punctatus*)

**DOI:** 10.3389/fimmu.2026.1763508

**Published:** 2026-05-07

**Authors:** Young Kyung Park, Fenny Patel, Hossam Abdelhamed, Larry A. Hanson, Mark L. Lawrence, Hasan C. Tekedar

**Affiliations:** Department of Comparative Biomedical Sciences, College of Veterinary Medicine, Mississippi State University, Mississippi State, MS, United States

**Keywords:** *Aeromonas hydrophila*, recombinant protein, Tad pilus, TadZ, type IV pili, type IVc pilus

## Abstract

*Aeromonas hydrophila* is a Gram-negative pathogen responsible for motile *Aeromonas* septicemia (MAS) in fish, particularly in catfish aquaculture. The emergence of virulent *A. hydrophila* (vAh) has intensified the need to identify promising immunogenic targets for disease control. In this study, we aimed to evaluate the immunogenic potential and *in vivo* relevance of recombinant TadZ, a conserved cytoplasmic pilus assembly protein essential for the tight adherence (tad) pilus system in virulent *A. hydrophila*. We generated a schematic model of the Tad pilus machinery and mapped operon organization across diverse bacteria, revealing its conserved architecture. Fish were immunized intraperitoneally with the 44 kDa TadZ protein purified by Ni-NTA chromatography and emulsified with adjuvant, and they were subsequently challenged with an LD_80_ dose of vAh. After 7 days post-challenge, TadZ immunization significantly reduced mortality compared to the non-immunized (NI) group, with mortality decreasing from 79.6% in the NI group to 26.1% in the TadZ-immunized group, corresponding to a relative percent survival (RPS) of 67.2%. Bacterial load analysis demonstrated a significant reduction in the liver of TadZ-vaccinated fish. Serum antibody analysis revealed significantly elevated IgM titers against both recombinant TadZ and heat-killed wild-type antigens after immunization and challenge, indicating robust humoral immune activation. These findings suggest that TadZ immunization induces antigen-specific antibody responses that may contribute to protection against vAh infection. Together, these findings identify TadZ as an immunogenic component of the Tad pilus system with *in vivo* relevance and support its further evaluation as a promising antigen for future vaccine development in catfish.

## 1 Introduction

*Aeromonas hydrophila* is a Gram-negative pathogen commonly found in freshwater environments. It is known for causing motile *Aeromonas* septicemia (MAS) in a wide range of fish (1–3). This disease often results in high mortality rates, posing a significant economic threat to the global aquaculture industry (4). It has occasionally been reported to infect humans as an opportunistic pathogen following exposure to contaminated water or aquatic animals (5, 6). In the southeastern United States, a particularly virulent clonal subgroup, represented by strain ML09-119, caused outbreaks of MAS in catfish aquaculture beginning in 2009, resulting in high mortalities and significant economic damage (7–9).

Antibiotics are approved for treatment of MAS in catfish aquaculture. However, potential emergence of multidrug-resistant pathogens is a global concern, so alternative preventative strategies are desirable (10, 11). Inactivated and live-attenuated vaccines offer limited protection, largely due to the high level of antigenic diversity among *A. hydrophila* strains (12). Therefore, identifying antigens that are both highly conserved and functionally essential is important for development of broadly protective recombinant vaccines (12, 13). Given these challenges, it is vital to determine key structural and virulence factors that are critical for pathogenesis of virulent *Aeromonas hydrophila* (vAh).

One unique virulence system in vAh is the tight adherence (tad) pilus, recently reclassified as a type IVc pilus (T4cP) (14). Tad pili mediate surface attachment, biofilm formation, and host colonization across diverse bacteria. They are essential for biofilm formation in *Aggregatibacter actinomycetemcomitans* (15) and enable plant root colonization in *Pseudomonas chlororaphis* (16). They also contribute to host invasion and serum resistance in *Vibrio vulnificus* and promote oyster colonization by increasing bacterial retention during feeding (17, 18). In vAh, the Tad operon encodes 13 proteins required for pilus assembly and anchoring at the bacterial pole, and its deletion impairs biofilm formation, reduces virulence, and alters surface morphology (19).

One promising target of the Tad pilus system in vAh is TadZ, a cytoplasmic ATPase-like protein involved in polar localization that plays both regulatory and structural roles in pilus biogenesis (20). Mutational analyses have shown that deviations in key conserved residues of TadZ, particularly within the Walker A motif, result in little or no ATPase activity while still retaining its function in polar localization (21, 22). Although not surface-exposed, TadZ is thought to anchor the Tad pilus secretion system at the bacterial pole and guide pilus assembly as a member of the MinD/ParA superfamily of ATPases, which regulate the spatial organization of cellular structures by binding to membranes and recruiting partner proteins in a nucleotide-dependent manner (23, 24).

Comparative genomic analysis revealed that TadZ is present in all evaluated *A. hydrophila* genomes, both vAh and non-vAh. In contrast, the rest of the Tad operon is generally found in vAh strains but largely missing or incomplete in most non-vAh isolates, with a few exceptions (19, 25). Beyond *A. hydrophila*, the Tad operon is broadly conserved across a wide range of Gram-negative bacteria, Actinobacteria, and even Archaea (26). Although TadZ is widely distributed, its contribution to virulence is primarily manifested when the complete operon is intact and functionally expressed, a feature characteristic of highly virulent vAh strains. This pattern emphasizes the central role of TadZ in pilus biogenesis and suggests its potential relevance as a target for disease control strategies, including vaccine development (25, 27).

In this study, we first investigated the conservation of TadZ across *A. hydrophila* strains and its functional role within the Tad pilus system. In addition, we cloned and expressed the recombinant TadZ protein and evaluated its potential as a recombinant immunogenic antigen against vAh. We examined whether TadZ could elicit specific antibody responses in channel catfish and provide protection against challenge with the virulent field isolate *A. hydrophila* ML09-119. Together, these findings support TadZ as a promising antigen candidate in pathogenic species harboring the Tad operon, emphasizing how conserved proteins could contribute to improving vaccine strategies not only for controlling motile *Aeromonas* septicemia but also for providing a conceptual framework for evaluating Tad-associated proteins in other bacterial species.

## 2 Materials and methods

### 2.1 Ethics statement

The animal study was approved by the Institutional Animal Care and Use Committee of Mississippi State University. The study was conducted in accordance with the local legislation and institutional requirements.

### 2.2 Structural prediction of the Tad pilus system

A schematic illustration of the Tad pilus assembly machinery was created using BioRender (https://www.biorender.com). The model integrates known structures and localization data from previously characterized Tad systems in *A. actinomycetemcomitans*, *Caulobacter crescentus*, and *P. aeruginosa* (23, 26, 28, 29). Each component, including Flp, RcpA, RcpB, RcpC, TadV, TadZ, TadA, TadB, TadC, TadD, TadE, TadF, and TadG, was manually positioned based on known or predicted localization (cytoplasmic, inner membrane, periplasmic, outer membrane, or extracellular) and conserved domain function.

### 2.3 Genomic distribution and structural organization of the Tad operon

We conducted a comparative analysis on the distribution of the Tad operon across 13 bacterial genera and 19 different pathogenic species to evaluate the localization of the TadZ homologs within the Tad operon. Protein sequences of interest were retrieved in FASTA format from the NCBI database, and corresponding genome assemblies were downloaded in GenBank format for downstream analysis. Genomic analyses were conducted using the TXSScan tool available on the Galaxy Pasteur platform (https://galaxy.pasteur.fr/, accessed May 2025). The TXSScan workflow (Galaxy Tool Version 1.0.0) was executed under the Genome Annotation section, specifying bacteria as the taxonomic group and using unordered replicons with default parameters. TXSScan output files were examined to identify predicted secretion system operons containing Tad components. The first gene of each putative operon was recorded, and surrounding genes associated with the Tad locus were identified based on their annotations and relative genomic positions. To visualize the gene organization of these operons, genome annotation files were imported into SnapGene (v6.2.1, GSL Biotech LLC). Identified genes were located using their locus tags or protein IDs, and operon structures were mapped manually, the operon organization was then schematically illustrated using BioRender (https://www.biorender.com). Gene diagrams were generated in SnapGene to accurately depict gene sizes and orientations, enabling comparative analysis of operon architectures across different species.

### 2.4 Bacterial strains and growth conditions

The bacterial strains and plasmids used in this study were *Escherichia coli* DH5α (Thermo Fisher Scientific, Waltham, MA, USA) for cloning and BL21(DE3) (Novagen, Madison, WI, USA) for protein expression, with the pET-28a(+) vector (Novagen). *A. hydrophila* was cultured in brain heart infusion (BHI) agar and broth (Difco, Sparks, MD, USA) at 30 °C, and *E. coli* strains were cultured in Luria–Bertani (LB) agar and broth (Difco) with 50 µg/ml kanamycin sulfate (MilliporeSigma, Burlington, MA, USA; Cat. 60615) at 37 °C.

### 2.5 Plasmid construction and cloning

The *tadZ* gene region of vAh ML09–119 was amplified with gene-specific primers (F: GAATTCATGAGTGAACAAGTCAAACAG; R: AAGCTTGCGCCTCCCGCCCCGGACTCG) and subsequently cloned into the pET-28a(+) expression vector. The amplified product was purified using a PCR purification kit (IBI Scientific, Dubuque, IA, USA). The pET-28a(+) vector and the purified PCR product were both digested with EcoRI and HindIII (New England Biolabs, Ipswich, MA, USA) at 37 °C for 4 hours. The digested products were purified and ligated using T4 DNA ligase (NEB) at 16 °C overnight. The ligation mixture was transformed into chemically competent *E. coli* DH5α cells using heat-shock at 42 °C for 60 seconds and recovered in 100 μL of LB medium at 37 °C for 1 hour. Colonies were selected on LB agar plates supplemented with 50 μg/ml kanamycin sulfate (MilliporeSigma), and positive clones were screened by colony PCR. Plasmid DNA was extracted using a plasmid mini prep kit (IBI) and verified by restriction enzyme digestion. The confirmed recombinant plasmid was then transformed into *E. coli* DH5α cells for plasmid propagation. Positive clones were screened by colony PCR and confirmed by restriction enzyme digestion.

### 2.6 Recombinant protein expression

The verified recombinant plasmid was transformed into *E. coli* BL21(DE3) and stored at –80 °C in 80% glycerol stocks for subsequent expression and purification. For small-scale expression, a single colony harboring the recombinant pET-28a(+) plasmid was inoculated into 5 mL of LB medium with kanamycin and cultured at 37 °C with shaking at 200 rpm until the optical density at 600 nm (OD_600_) reached 0.6. Protein expression was induced by adding 1 mM isopropyl β-D-1-thiogalactopyranoside (IPTG; Invitrogen™, Thermo Fisher Scientific, Waltham, MA, USA; Cat. No. AM9462), followed by incubation at 30 °C for 8 hours with shaking. Large-scale expressions were performed under the same conditions in 50 mL LB-kanamycin medium. For SDS-PAGE analysis, 1 mL of whole cell lysate was spinned down and the pellet was mixed with 4x Laemmli sample buffer (Bio-Rad, Hercules, CA, USA, Cat. No. 1610747) supplemented with 2-mercaptoethanol (Thermo Fisher Scientific, Cat. No. 21985023) and heated at 95 °C for 10 min. Proteins were separated on 12% SDS-polyacrylamide gels at 120 V for 1 h 30 min using a PROTEAN^®^ II XL Cell electrophoresis system (Bio-Rad) and visualized by Coomassie Blue staining. Gel images were acquired with the ChemiDoc™ MP imaging system (Bio-Rad, Hercules, CA, USA) using Image Lab™ Touch Software, version 2.4.0.03, with optimal auto-exposure setting. Whole bacterial lysates from untransformed *E. coli* BL21 (DE3) and uninduced *E. coli* BL21 (DE3) harboring the recombinant plasmid served as controls.

### 2.7 Purification of recombinant TadZ protein

Recombinant proteins were expressed in *E. coli* BL21 (DE3) cells harboring the recombinant plasmid. Cells were harvested at 6,000 × g for 20 min at 4 °C and pellets were resuspended in 4 mL STE (NaCl–Tris–EDTA). The mixture was supplemented with 10 mL of 1 M DL-dithiothreitol (DTT; Sigma-Aldrich, St. Louis, MO, USA; Cat. No. D5545) and N-lauroylsarcosine sodium salt (Sigma-Aldrich, St. Louis, MO, USA; Cat. No. L5125) to 0.5-0.6% w/v final and incubated on ice for 2 min, followed by the addition of STE to 10 mL. Cells were disrupted by sonication on ice (4 × 20 s), and the lysates were centrifuged at 6,000 × g for 20 min at 4 °C. Pellets were resuspended in 4 mL of 6 M guanidine-HCl (Sigma-Aldrich; Cat. No. 50950) and sonicated on ice (2 × 10 s). After centrifugation at 6,000 × g for 20 min at 4 °C, supernatants were passed through a 0.45-μm syringe filter. For SDS-PAGE, cell pellets were boiled for 5 min with Laemmli loading buffer (Bio-Rad) containing 5% 2-mercaptoethanol (Thermo Fisher Scientific).

TadZ protein was purified by Ni-NTA affinity chromatography using Ni-Indigo resin (Cube Biotech, Wayne, PA, USA; Cat. No. 75103). Filtered and equilibrated lysate was loaded onto a column equilibrated with binding buffer (20 mM Tris–HCl, 500 mM NaCl, and 5 mM imidazole, 6 M Urea, 1 mM 2-mercaptoethanol pH 8.0) and 10 ml of wash buffer (20 mM Tris–HCl, 500 mM NaCl, and 20 mM imidazole, 6 M Urea, 1 mM 2-mercaptoethanol pH 8.0), then eluted with 5 ml of elution buffer (20 mM Tris–HCl, 500 mM NaCl, and 500 mM imidazole, 6 M Urea, 1 mM 2-mercaptoethanol, pH 8.0). Eluate was buffer exchanged into PBS using Amicon^®^ Ultra 10K (MilliporeSigma; Cat. No. UFC501024) to a final volume of 2–3 mL. Protein concentrations were measured at A280 on a Nanodrop (Thermo Fisher Scientific). Purity was assessed by 12% SDS-PAGE with Coomassie staining, and the protein was stored at −80 °C.

### 2.8 Immunoblot analysis

Purified TadZ (1 µg/lane) was separated on 12% SDS-PAGE and transferred to a polyvinylidene difluoride (PVDF) membrane (Bio-Rad Laboratories, Hercules, CA, USA). The membrane was blocked in Tris-buffered saline with Tween-20 (TBST) buffer (10 mM Tris-HCl, 150 mM NaCl, 0.1% Tween-20, pH 8.0) containing 5% nonfat dry milk (Bio-Rad) for 1 h at room temperature, then incubated with mouse monoclonal anti-6x-His tag antibody (1:10,000; Invitrogen, Cat. No. MA1-21315) overnight at 4 °C. After 3 × TBST washes, secondary antibody was followed with horseradish peroxidase (HRP)-conjugated goat anti-mouse IgG (1:100,000; Invitrogen, Thermo Fisher Scientific, USA, Cat. No. 31431) for 1 h at room temperature. Membrane was developed using SuperSignal West Pico PLUS Chemiluminescent Substrate (Thermo Fisher Scientific, Cat. No. 34577).

### 2.9 Fish challenge

Pathogen-free channel catfish fingerlings (n=160; mean weight: 61.95 g and 7.88 inches, respectively) were randomly stocked in sixteen 40 L tanks (10 fish/tank) and supplied with flow-through dechlorinated municipal water and continuous aeration. Fish were acclimated for 10 days and fed daily with commercial dry pellets. The aeration and water temperature (29 °C ± 0.2 °C) were monitored daily. ​In this study, 160 fish were divided into four groups to evaluate the immunogenicity and protective efficacy of TadZ protein. Fish were immunized intraperitoneally with a 30:70 (v/v) emulsion (150 µL) of TadZ stock (0.25 µg/µL) and Freund’s Complete Adjuvant (FCA; Sigma-Aldrich, F5881), delivering 11.25 µg TadZ per fish, while control groups received no injection (non-immunized, NI), PBS, or PBS emulsified with adjuvant (PBS-A). Three weeks post-immunization, all groups were challenged by bath immersion with vAh strain ML09-119. For immersion challenge, 225 mL of bacterial culture (1.45 × 10^9^ CFU/mL, by plate count) was added to 40 L of water, and fish were exposed for 6 hours. This challenge dose corresponded to the LD_80_ (lethal dose for 80% mortality) for channel catfish of similar size, as determined in preliminary experiments. Mortality was monitored for 7 days after the challenge, and relative percent survival (RPS) was calculated using the formula: RPS = [1 – (mortality in vaccinated group/mortality in control group)] × 100 (3, 30).

### 2.10 Quantification of bacterial quantities in organs

Fish were humanely euthanized two days post-challenge with a lethal dose of 500 mg/L ethyl 3-aminobenzoate methanesulfonate (MS-222; Sigma-Aldrich, Cat. No. E10521). Three major organs, the anterior kidney, liver, and spleen, were aseptically removed. To quantify bacterial concentrations, whole organ samples were homogenized with pestles, and serial dilutions (10^−1^, 10^−2^, and 10^−3^) were performed in sterile PBS. From each dilution, 100 µL was spread onto separate BHI agar plates and incubated at 30 °C for 24 h. Colonies were counted on plates exhibiting 30 to 300 colonies to ensure statistical reliability. The CFU values were transformed by adding 1 to each count and then applying a base-10 logarithm, reported as log_10_ (CFU + 1) per gram of tissue.

### 2.11 Serum antibody determination

Blood samples were collected as described previously (3). Briefly, blood was collected from the caudal vein of 4 fish per group in untreated tubes before and after immunization under MS-222 anesthesia prior to organ harvesting to evaluate systemic antibody responses. After clotting overnight at 4 °C, serum was separated by centrifugation at 3,000 × g for 10 min at 4 °C, pooled, aliquoted (50 µl per group), and stored at -80 °C. For ELISA, 96-well high-binding microplates (Nunc, Thermo Fisher Scientific, USA) were coated overnight at 4 °C with 100 µl of antigen in coating buffer (0.037 M citric acid, 0.158 M Na_2_HPO_4_, pH 6.0). Antigens included a suspension of heat-killed vAh strain ML09-119, prepared by heating at 65 °C for 1 hour at 5 × 10^7^ CFU/ml, and purified TadZ protein (0.25 µg/µl). Plates were washed three times with PBS containing 0.05% (v/v) Tween 20 (PBST), then blocked with 125 µl of 1% (w/v) bovine serum albumin (BSA; Sigma-Aldrich, Cat. No. A9418) in PBST for 1 hour at room temperature. After three washes, diluted fish serum (1:20) was added (100 µl/well) and incubated at 37 °C for 2 hours, followed by three washes with PBST. Next, 100 µl of monoclonal antibody 9E1 (anti-catfish IgM, 1:100 dilution (31); was added and incubated at 37 °C for 1 hour. After washing, 100 µl of HRP-conjugated goat anti-mouse IgG secondary antibody (1:10,000 dilution; Invitrogen) was added and incubated at room temperature for 1 hour. Plates were washed three times, then incubated with 100 µl of TMB substrate (Thermo Scientific™, Cat. No. 34024) for 15–30 min. The reaction was stopped with 50 µl of 2 M H_2_SO_4_, and absorbance at 450 nm was measured using a microplate reader (Gen5, BioTek, USA). Control wells containing PBS instead of serum were included on each plate. To standardize the results, the average background absorbance from control wells was subtracted from the absorbance values of each sample.

### 2.12 Statistical analysis

Data analysis was performed using IBM SPSS Statistics (Version 29.0; IBM Corp., Armonk, NY, USA), and graphs were generated using GraphPad Prism (Version 10.0.0 for Windows; GraphPad Software, Boston, MA, USA). Survival data were analyzed using Kaplan–Meier survival estimation, and differences among groups were assessed using the log-rank (Mantel–Cox) test followed by pairwise log-rank comparisons. One-way analysis of variance (ANOVA) was conducted to evaluate differences in mortality rates. A univariate general linear model (GLM) was applied to assess viable cell concentrations and serum antibody levels across experimental groups. *Post hoc* one-way ANOVA tests (Tukey’s multiple comparison) were subsequently performed to identify specific group differences. Data are presented as means ± standard error (SEM), and *p*-values of <0.05, <0.01, and <0.001 were considered statistically significant.

## 3 Results

### 3.1 Structural organization of the Tad pilus system in vAh strain ML09-119

**Figure 1:**
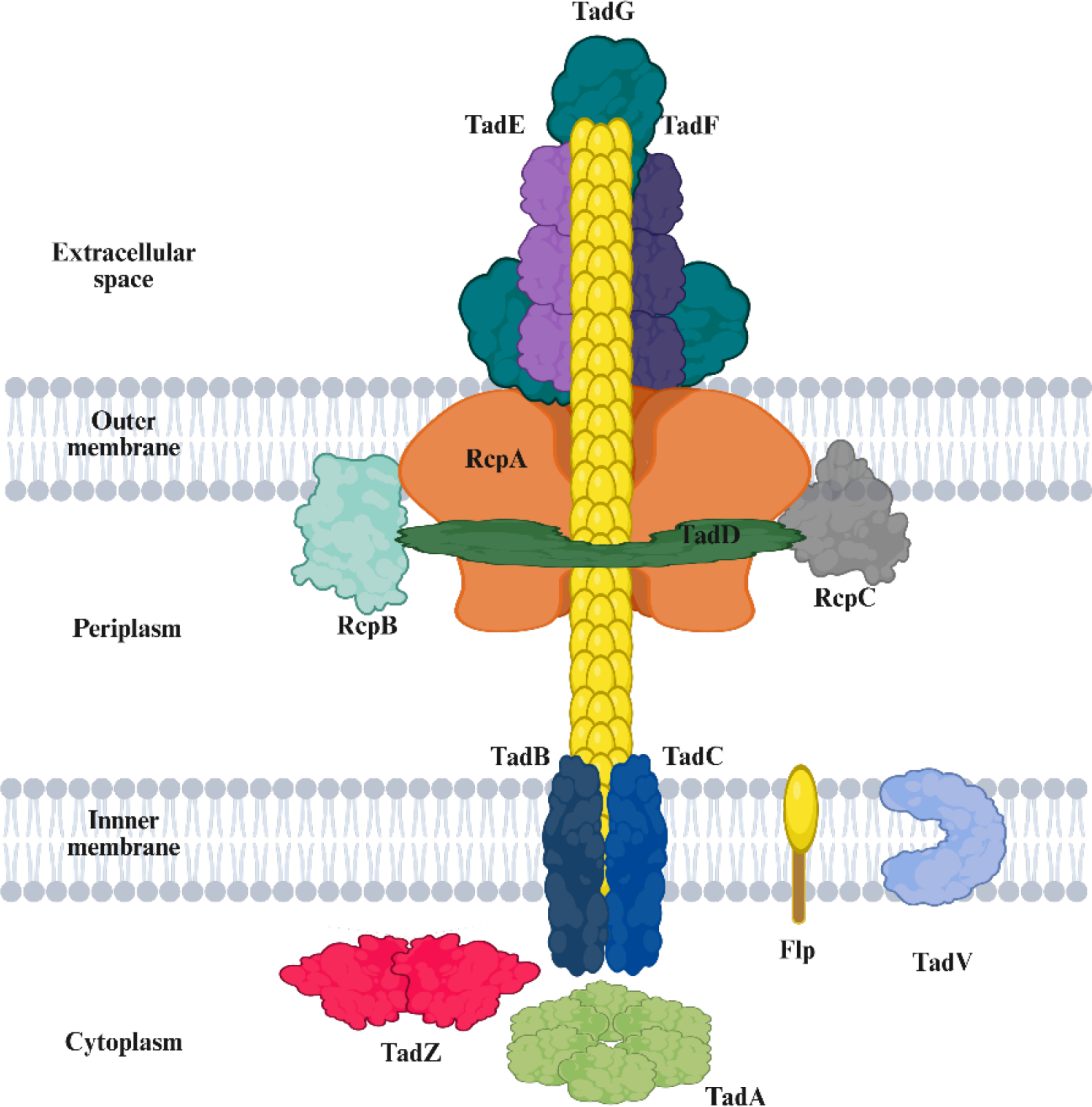
Diagram of a bacterial tight adherence (Tad) pilus system spanning extracellular space, outer membrane, periplasm, inner membrane, and cytoplasm, with labeled protein components including Flp, RcpA, RcpB, RcpC, TadV, TadZ, TadA, TadB, TadC, TadD, TadE, TadF, and TadG illustrating their locations.

To provide a conceptual overview of the Tad pilus machinery in vAh, we generated a schematic model based on the previously characterized Tad systems (20, 23, 26, 29). This schematic model showed a multi-protein complex that spans the inner membrane, periplasm, and outer membrane, ultimately projecting a polymerized Flp pilus fiber into the extracellular space. As shown in **Figure 1**, TadZ is in the cytoplasm adjacent to TadA, an ATPase known to power the extrusion of Flp subunits through the inner membrane platform formed by TadB and TadC. TadV, a membrane-associated prepilin peptidase, was positioned close to the Flp pilin to represent its critical role in cleaving Flp before it assembles into a pilus. The Flp subunit, depicted as the main pilus filament, was illustrated extending through the outer membrane channel formed by RcpA, which facilitates its surface exposure. Periplasmic components such as RcpB and TadD are thought to stabilize the pilus as it traverses the cell envelope, while outer membrane and tip-associated proteins including TadE, TadF, and TadG may contribute to pilus maturation and host interactions.

### 3.2 Genomic comparison of Tad operons across bacterial strains

To explore conservation of the Tad operon, we analyzed 23 bacterial genomes representing 19 unique strains from 19 species across 13 genera, selected for their ecological and pathogenic relevance. These included aquatic pathogens such as *A. hydrophila* ML09-119, *A. salmonicida* A449, and *A. veronii* B565, as well as the fish-associated *Vibrio vulnificus* CMCP6 I and *V. fischeri* ES114, both possessing two chromosomes. Also examined was *Burkholderia pseudomallei* K96243, a zoonotic pathogen with two chromosomal Tad loci, and *Pasteurella multocida* subsp. *multocida* Pm70, an avian pathogen with zoonotic potential. Human-associated bacteria were also represented, including *A. actinomycetemcomitans* HK1651, *Haemophilus ducreyi* 35000HP, *Yersinia enterocolitica* subsp. *palearctica* 105.5R(r), and *Y. pseudotuberculosis* IP 31758. Environmental or plant-associated species included *Caulobacter crescentus* CB15, *C. vibrioides* CB13b1a, *Rhodopseudomonas palustris* CGA009, and *Pectobacterium atrosepticum* JG10-08. Additional strains included *Pseudomonas aeruginosa* PAO1, *Bordetella pertussis* Tohama, *B. parapertussis* 12822, and the gut-associated *Bifidobacterium breve* ACS-071-V-Sch8b. These strains represent diverse ecological niches and provide a basis for a comprehensive strain-level assessment of tad operon distribution (26, 29, 32).

**Figure 2:**
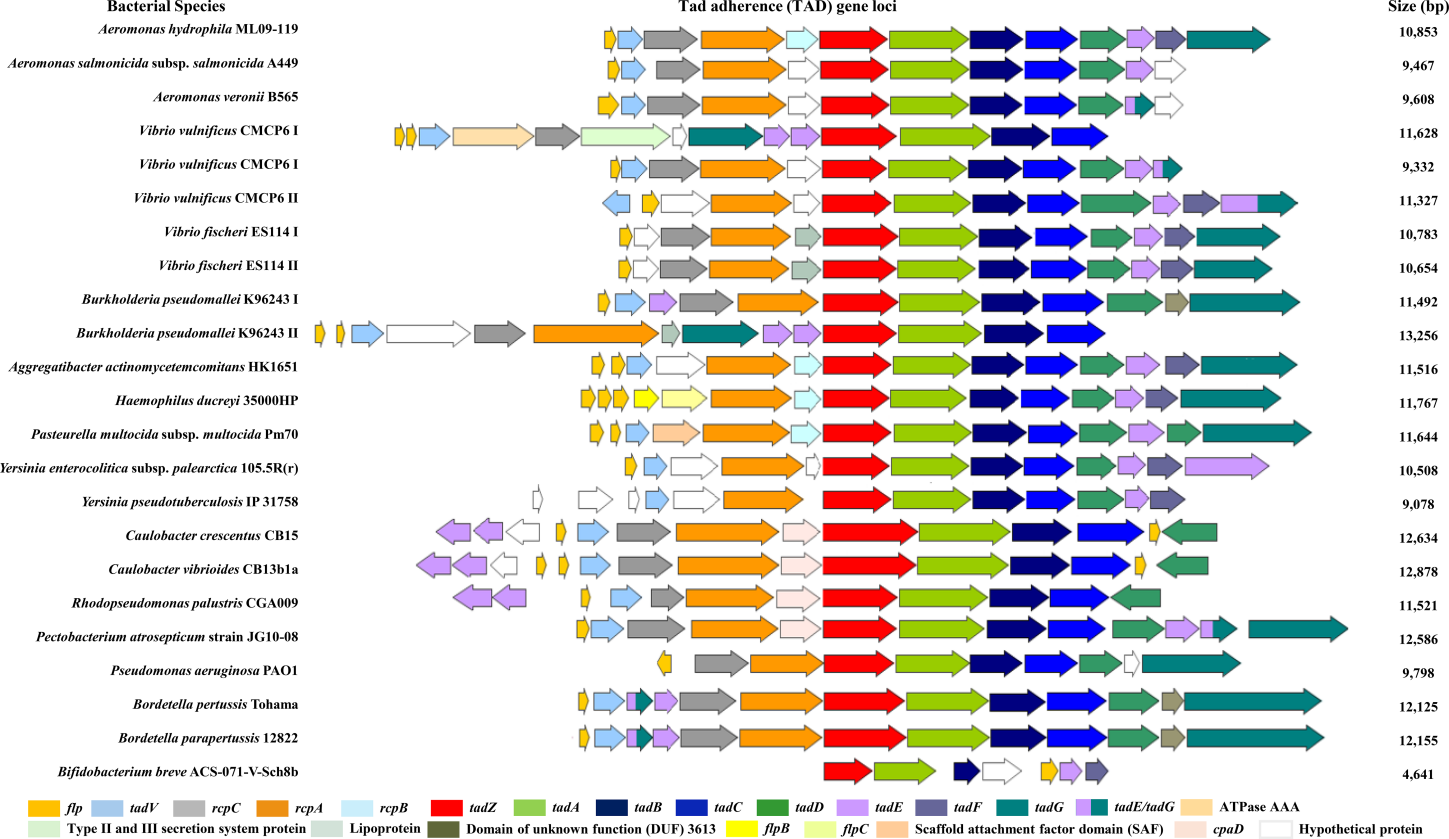
Gene cluster diagram comparing tight adherence (Tad) gene loci and gene orientations among multiple bacterial species, with arrows color-coded to indicate gene locations. Each row shows a bacterial strain and corresponding gene arrangement, alongside gene cluster size in base pairs.

As shown in **Figure 2**, the Tad locus is fundamentally composed of 12–15 genes, although variation exists in gene order, orientation, and total number across strains. This overall organization is consistent with prior descriptions of Tad operons across diverse bacterial species (26, 29, 32). Among the 19 strains analyzed, more than half exhibited a highly similar gene organization. Most strains (18 of 19) possessed the core Tad components, including *flp*, *rcpA*, *tadZ*, *tadA*, *tadB*, and *tadC*, except for *Bifidobacterium breve* ACS-071-V-Sch8b, which is relatively small and lacked *rcpA*. A complete Tad operon was observed in most species, including *A. actinomycetemcomitans* HK1651, *A. hydrophila* ML09-119, *A. salmonicida* A449, *A. veronii* B565, *B. pseudomallei* K96243 (Chromosome I), *H. ducreyi* 35000HP, *P. atrosepticum* JG10-08, *P. multocida* Pm70, *V. fischeri* ES114 (Chromosome II), *V. vulnificus* CMCP6 (Chromosome II), and *Y. enterocolitica* 105.5R(r). These operons consisted of 12–15 genes arranged in a tightly clustered and co-directional manner, resembling the canonical Tad system first described in *A. actinomycetemcomitans* (26, 32). In contrast, *Caulobacter* species and *R. palustris* CGA009 exhibited different operon arrangements, with accessory genes such as *tadE* and *tadG* located upstream of the core components. *V. vulnificus* CMCP6 also carried a second, more fragmented Tad operon on Chromosome I, with *tadE* and *tadG* positioned centrally. Despite these structural differences, the conserved core block consisting of *tadZ*, *tadA*, *tadB*, and *tadC* was retained across nearly all strains examined, highlighting strong evolutionary conservation of the central Tad assembly machinery. Accession numbers, Tad locus and genome sizes, for all strains analyzed are summarized in **Supplementary Table 1**.

### 3.3 Cloning, purification, and verification of TadZ protein

**Figure 3:**
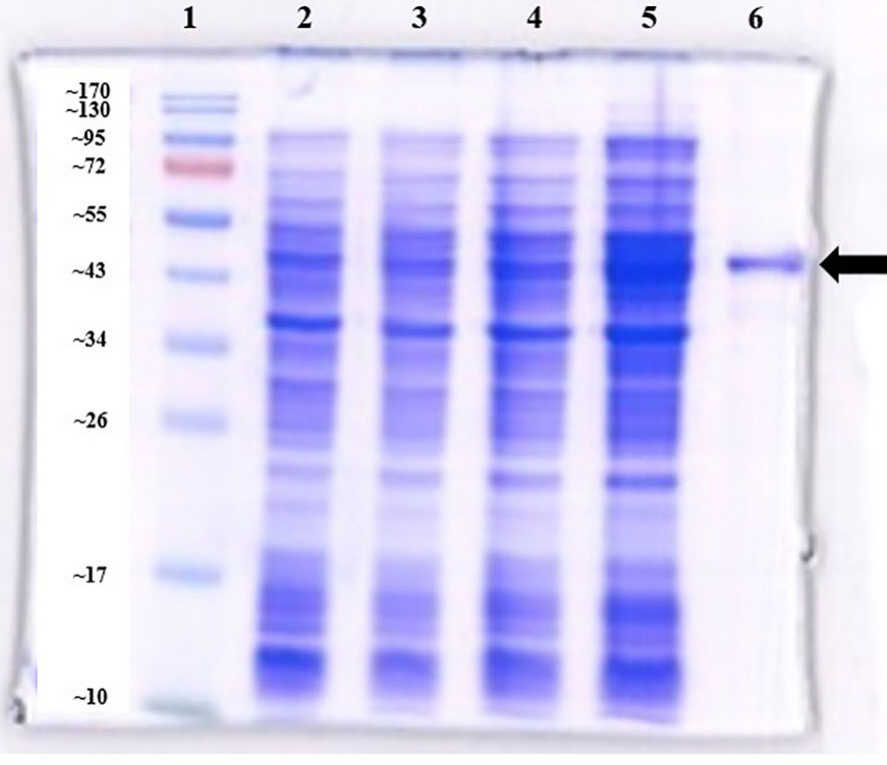
SDS-PAGE analysis of TadZ protein expression and purification. Samples were separated on a 12% SDS-PAGE gel and stained with Coomassie Brilliant Blue. Lane 1: Prestained protein marker; Lane 2: *E. coli* BL21(DE3) uninduced; Lane 3: *E. coli* BL21(DE3) induced with 1 mM IPTG at 30 °C for 8 hours; Lane 4: Uninduced BL21(DE3) pET-28a(+)-TadZ at 30 °C for 8 hours; Lane 5: Induced BL21(DE3) pET-28a(+)-TadZ with 1 mM IPTG at 30 °C for 8 hours; Lane 6: Purified TadZ protein. A strong band near ~44 kDa was observed in lane 5 compared to lane 4, and the band in lane 6 corresponded to the expected size of the His-tagged TadZ protein.

**Figure 4:**
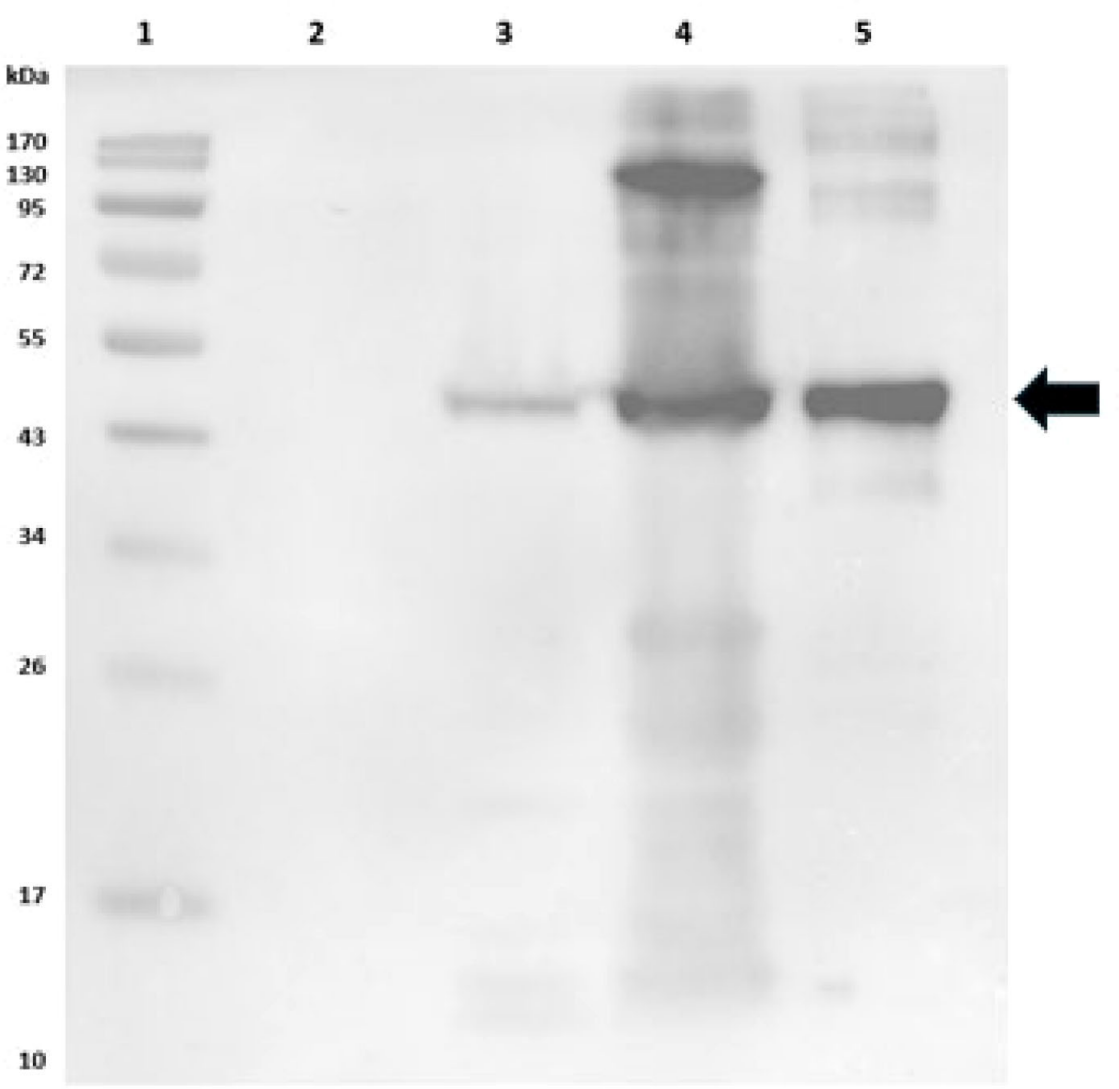
Proteins were separated on a 12% SDS-PAGE gel and transferred to a PVDF membrane. The membrane was blocked with 5% non-fat dry milk in TBST and probed with mouse monoclonal anti-6×His antibody (1:10,000), followed by HRP-conjugated goat anti-mouse IgG secondary antibody (1:100,000). Lane 1: Molecular weight marker; Lane 2: *E. coli* BL21(DE3) without plasmid; Lane 3: Uninduced lysate of *E. coli* BL21(DE3) harboring pET-28a(+)-*tadZ*; Lane 4: Induced lysate of *E. coli* BL21(DE3) harboring pET-28a(+)-*tadZ*; Lane 5: Purified recombinant TadZ protein.

The predicted molecular weight, including the N-terminal His-tag and vector-derived residues, was ~43.7 kDa. SDS-PAGE revealed a distinct band at ~44 kDa (**Figure 3**), with strong expression observed in induced lysates (lane 5) compared with uninduced cells (lane 4) and vector controls (lanes 2–3). Western blotting with an anti-His monoclonal antibody confirmed the identity of TadZ, showing a strong immunoreactive band at ~44 kDa in both induced lysates and purified protein (**Figure 4**; lanes 4–5). A faint band was observed in the uninduced lysate (lane 3), while no signal was detected in the BL21(DE3) negative control (lane 2), confirming antibody specificity. Following confirmation of TadZ expression and purity, we next evaluated its *in vivo* relevance using survival, bacterial burden, and immune response measures. TadZ immunization was associated with improved survival, reduced bacterial burdens in target organs, and enhanced antigen-specific antibody responses following acute vAh challenge.

### 3.4 *In vivo* protective effects of TadZ immunization

**Figure 5:**
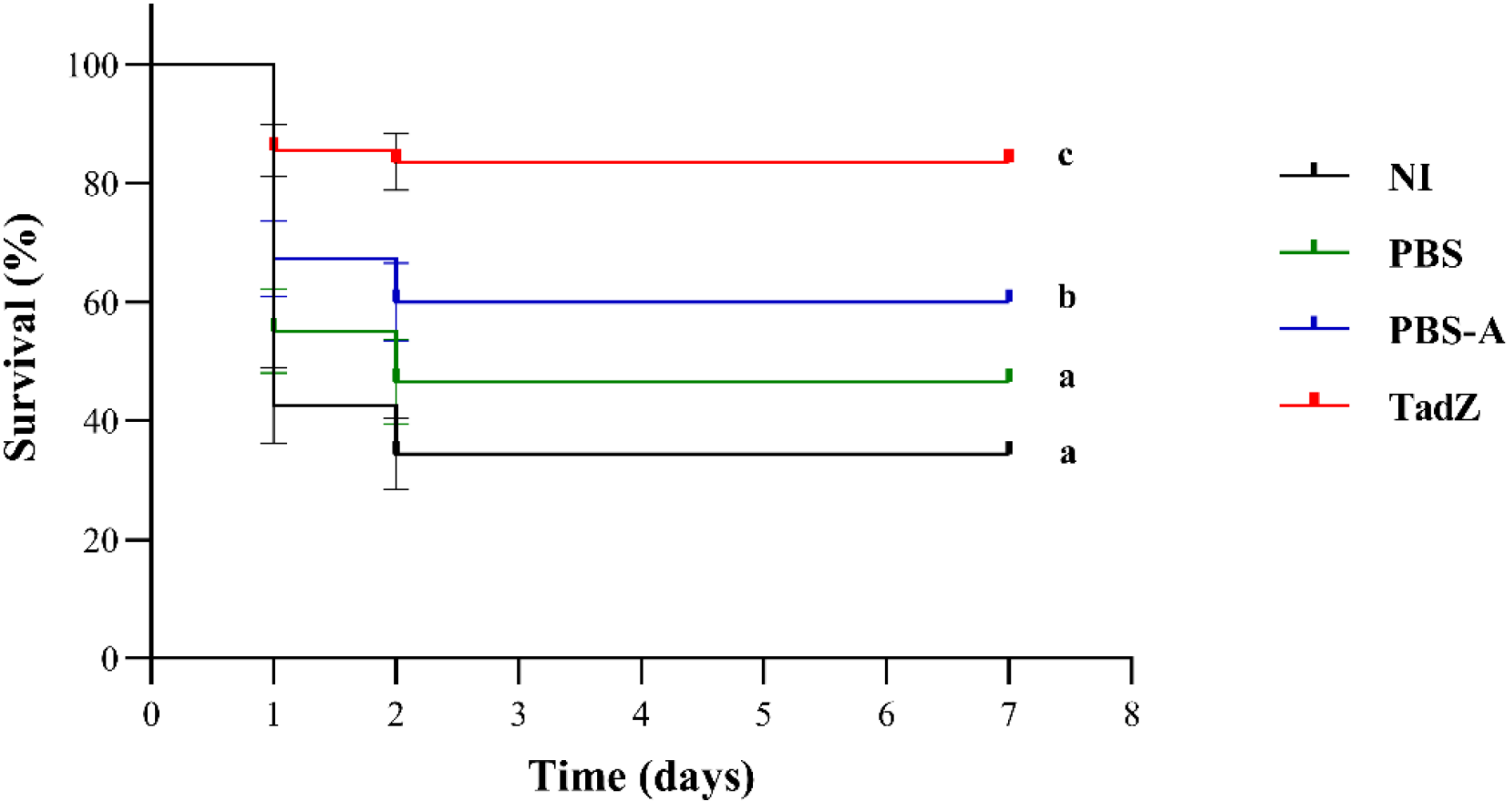
Kaplan–Meier survival of channel catfish immunized with TadZ recombinant protein following challenge with virulent *Aeromonas hydrophila* ML09-119. Channel catfish fingerlings (n=160) were divided into four groups (10 fish per tank, 4 replicate tanks per group): NI (non-immunized), PBS (phosphate-buffered saline), PBS-A (PBS with adjuvant), and TadZ (TadZ protein with adjuvant). Three weeks post-immunization, fish were challenged by bath immersion with *A. hydrophila* ML09–119 at an LD_80_ dose. Survival was monitored over 7 days post-challenge. Kaplan–Meier analysis revealed significant differences among groups (log-rank test, χ^2^ = 24.01, *p* < 0.0001). Pairwise log-rank comparisons showed that the TadZ-vaccinated group had significantly higher survival than NI and PBS controls (*p* < 0.001) and higher survival than PBS-A (*p* < 0.05). Groups sharing the same letter are not significantly different (pairwise log-rank test). Error bars represent standard error of the survival proportion.

To evaluate the *in vivo* relevance of TadZ vaccination against vAh infection, mortality was monitored for 7 days post-challenge and analyzed using Kaplan–Meier survival curves (**Figure 5**). Following immersion challenge, fish in the NI and PBS groups exhibited typical signs consistent with motile *Aeromonas* septicemia, including lethargy, reduced feeding, and occasional external hemorrhagic lesions, whereas these signs were less frequent in the TadZ-immunized group. The TadZ-immunized group showed the highest survival and the lowest cumulative mortality (26.1%), corresponding to an RPS of 67.2%. In contrast, the NI and PBS groups exhibited high mortalities of 79.6% and 73.1%, respectively, while the PBS-A group showed an intermediate mortality of 56.2%. Kaplan–Meier analysis indicated significant differences among groups (log-rank test, *p* < 0.0001). Pairwise log-rank tests showed that TadZ immunization resulted in significantly higher survival than NI and PBS (both *p* < 0.001) and higher survival than PBS-A (*p* = 0.021). PBS-A showed improved survival compared with NI (*p* = 0.015), whereas PBS did not differ significantly from NI (*p* = 0.426). One-way ANOVA of mortality proportions confirmed that TadZ immunization significantly reduced mortality compared with NI and PBS controls (*p* < 0.01).

### 3.5 Effect of TadZ immunization on bacterial quantities following vAh infection

**Figure 6:**
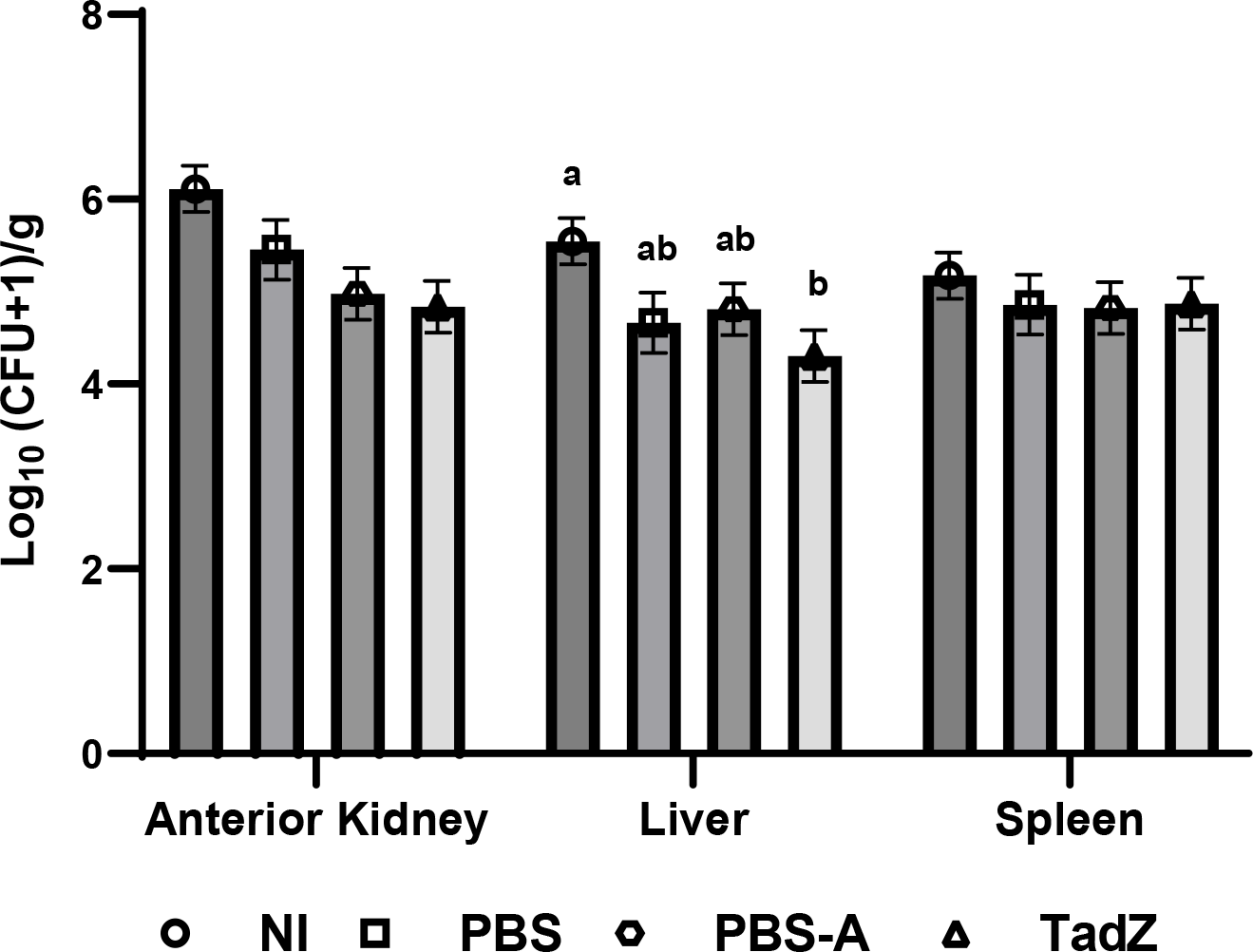
Quantification of bacterial loads in internal organs following *Aeromonas hydrophila* ML09-119 challenge. Bacterial loads were measured in the anterior kidney, liver, and spleen of channel catfish two days post-challenge with *A. hydrophila* ML09-119. Fish were assigned to four treatment groups: non-immunized (NI; ○), PBS (□), PBS with adjuvant (PBS-A; ⎔), and TadZ (▵). Organ samples were homogenized, serially diluted, and plated on BHI agar. Colony-forming units (CFU) were counted after 24–48 h of incubation at 30 °C. Values are expressed as mean log_10_(CFU + 1)/g of tissue ± SEM (n = 5 per group). A significant reduction in bacterial load was observed in the liver of the TadZ group compared to the NI group (*p* < 0.05, one-way ANOVA with Tukey’s *post-hoc* test). No statistically significant differences were observed in the anterior kidney or spleen.

Following survival analysis, to further characterize infection severity, bacterial concentrations were quantified in the anterior kidney, liver, and spleen of challenged fish (**Figure 6**). Overall, the TadZ-immunized group exhibited reduced bacterial concentrations in all three organs compared to the NI, PBS, and PBS-A groups. In the anterior kidney, the mean bacterial concentration was lowest in the TadZ group (4.84 ± 0.28 log_10_ CFU/g), and highest in the NI group (6.11 ± 0.25), followed by the PBS group (5.46 ± 0.32), PBS-A (4.98 ± 0.28). Although the reduction in the TadZ group did not show statistical significance compared to the other groups, a downward trend was observed. In the liver, the TadZ group showed a significantly lower bacterial concentration (4.30 ± 0.28 log_10_ CFU/g) compared to the NI (5.55 ± 0.25; *p* < 0.05). PBS (4.66 ± 0.32) and PBS-A (4.81 ± 0.28) exhibited intermediate bacterial concentrations, not significantly different from NI or TadZ groups. In the spleen, bacterial concentrations were similar across all groups, ranging from 4.82 to 4.87 log_10_ CFU/g, with no statistically significant differences observed (*p* > 0.05).

### 3.6 Analysis of serum antibody response

**Figure 7:**
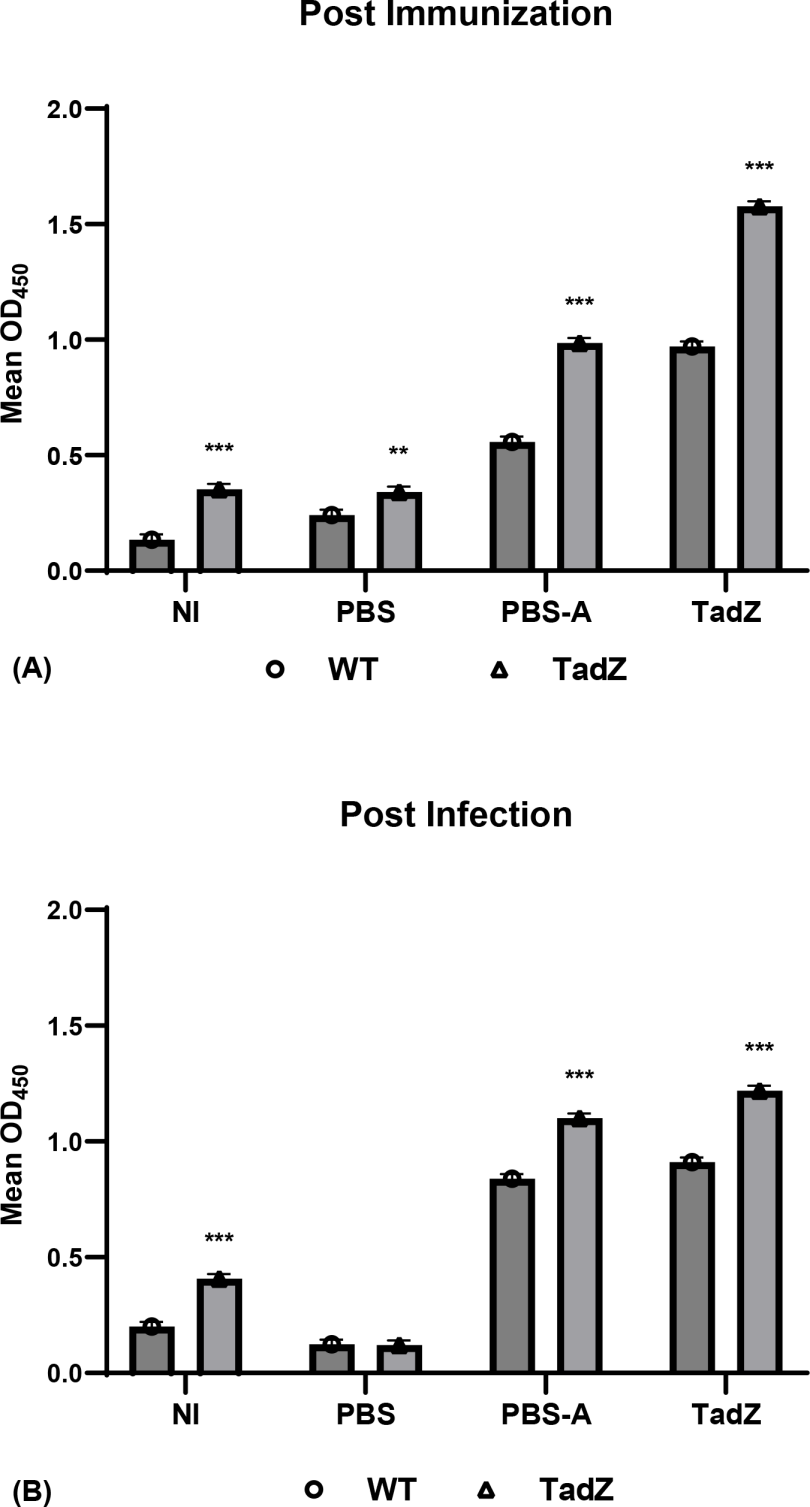
Serum antibody responses to wild-type antigens and TadZ in immunized channel catfish. ELISA was performed to assess antigen-specific serum IgM antibody responses at two time points: **(A)** three weeks post-immunization and **(B)** two days post-infection. Fish (n = 10/group) were immunized with PBS, PBS with adjuvant (PBS-A), or recombinant TadZ protein with adjuvant. Serum samples were pooled by group and tested against heat-killed wild-type *Aeromonas hydrophila* ML09-119 (WT; open circles, ○) or purified recombinant TadZ protein (open triangles, ▵). Plates were coated with antigen, and bound IgM was detected using monoclonal anti-catfish IgM antibody 9E1, followed by HRP-conjugated secondary antibody. OD_450_ values are presented as mean ± SEM. At both time points, TadZ-immunized fish showed significantly higher antibody levels against both WT and TadZ antigens compared to control groups. ***p < 0.001; **p < 0.01 (one-way ANOVA followed by Tukey’s *post-hoc* test). Symbols represent antigen type: WT (○), and TadZ (▵).

To assess whether the observed protection was associated with a humoral immune response, serum antibody levels were measured by indirect ELISA. Serum antibody titers against wild-type heat-inactivated *A. hydrophila* ML09-119 (WT) and recombinant TadZ protein were measured by indirect ELISA. The TadZ-vaccinated group showed the highest antibody levels (0.970 against WT and 1.577 against TadZ), which was significantly higher than all control treatments (*p* < 0.001 for NI and PBS-A, *p* < 0.01 for PBS). The PBS-A group exhibited intermediate titers (0.558 against WT and 0.985 against TadZ), while NI and PBS groups showed low responses (*p ≤*0.353). Following vAh challenge, antibody titers remained highest in the TadZ group (0.910 against WT and 1.220 against TadZ). Antibody titers were also elevated in the PBS-A group (0.839 and 1.101), compared to consistently low titers in NI and PBS groups. Statistical analysis confirmed significant differences among NI, PBS-A, and TadZ groups (*p* < 0.001), whereas PBS showed no significant change (**Figure 7**).

## 4 Discussion

*A. hydrophila* remains a major aquaculture pathogen, with the vAh clonal subgroup, represented by ML09-119, causing high mortality in catfish (4, 33). Among its virulence determinants, the Tad pilus system, referred to as the type IVc pilus (T4cP), is consistently present in vAh strains and plays a critical role in pathogenesis (29). The tad locus, first described as a widespread colonization island across diverse bacteria (26, 32), has since been recognized as an essential element for adhesion, colonization, biofilm formation, and host–pathogen interactions (29, 34). It was initially discovered in *A. actinomycetemcomitans*, where it was shown to be required for tight adhesion to surfaces (29, 32). Subsequent studies have revealed its presence in a broad range of environmental, commensal, and pathogenic bacteria, including *P. aeruginosa*, *C. crescentus*, and *A. hydrophila* (19, 26, 35). While the Tad system has been well characterized in a limited number of species, comparatively little work has been done in *Aeromonas* spp., and even less attention has focused on the roles of individual components such as TadZ.

Unlike other T4P systems, T4cP includes TadZ, a cytoplasmic localization factor that anchors the pilus assembly complex at the bacterial pole, Xu et al. (23) demonstrated that polar localization is vital for proper pilus function, and Perez-Cheeks et al. (22) further showed that TadZ is required for this localization of the Tad apparatus (22, 23). Ellison and Whitfield (34) also noted that such localization enhances both assembly efficiency and the stability of bacterial surface attachment. Based on these findings, we focused on TadZ because of its essential role in organizing the Tad pilus system (34).

To provide context for our results, we first considered the schematic organization of the Tad locus in vAh strains (4, 19). Based on previous studies, we inferred the structural arrangement of the vAh Tad system as a reference framework, in which TadZ is positioned adjacent to TadA in the cytoplasm and contributes to initiating and localizing the assembly machinery (22, 23, 26, 29, 32). In *A. actinomycetemcomitans*, TadZ has been described as a MinD-like spatial regulator that defines the site of Flp pilus biogenesis and is essential for tight adherence and virulence (32). Similarly, in vAh, TadZ appears to function as an ATPase-like cytoplasmic organizer required for assembly and function of the Tad pilus system, contributing directly to adhesion, biofilm formation, and virulence in catfish (19). Together, these findings suggest that TadZ is not limited to anchoring pili at the bacterial pole but also coordinating efficient pilus assembly, thereby enhancing stable attachment and promoting disease progression. Consistent with this, disruption of the Tad system leads to reduced adhesion in *A. actinomycetemcomitans* and diminished biofilm formation and virulence in vAh (19, 32).

Building on this, we compared the organization of Tad gene loci across diverse bacterial species. The analysis revealed varied operon structures among 23 strains from aquatic, environmental, zoonotic, and clinical sources. Despite differences in gene order and arrangement, a conserved block of *tadZ*, *tadA, tadB*, and *tadC* was consistently present. Most operons resembled the canonical Tad system described in *A. actinomycetemcomitans* (26, 29), suggesting a shared functional role in pilus assembly across species (34).

Aquatic pathogens such as *A. hydrophila* ML09-119, *V. vulnificus* CMCP6, and *V. fischeri* ES114 maintained complete operons with well-ordered, co-directional genes. The operon in *A. hydrophila* ML09–119 was particularly notable, containing the full Tad complement in an arrangement closely resembling that of *A. actinomycetemcomitans*, pointing to possible evolutionary conservation or horizontal gene transfer (HGT) (26). In contrast, *B. pseudomallei* K96243 encoded two distinct Tad operons on separate chromosomes, with one Tad-like cluster located on chromosome I and a canonical operon on chromosome II, suggesting distinct functional roles rather than simple duplication (36). Similarly, *C. crescentus* CB15 and *R. palustris* CGA009 contained full operons but with rearranged gene orders, in which *tadE* and *tad*G were located upstream while the core *tadZ*–*tadA*–*tadB*–*tadC* block remained intact. A comparable variation was observed in *V. vulnificus* CMCP6 chromosome I, where *tadE* and *tadG* appeared internally, disrupting the typical linear arrangement. Such modular flexibility may reflect species-specific regulatory adaptations or HGT events followed by local rearrangements (29, 37).

While bacterial adhesion is a key virulence mechanism in pathogens, in commensal or probiotic species it plays a beneficial role by supporting stable colonization and maintaining the host microbiome. Notably, probiotic species such as *Bifidobacterium breve* ACS-071-V-Sch8b also harbor intact Tad operons, indicating that the system can function beyond pathogenic contexts to promote beneficial epithelial colonization and biofilm formation (38, 39). Thus, traits linked to infection in pathogens (19, 26, 32) may, in other contexts, support persistence and symbiosis in beneficial microbes. Despite variation in operon size, orientation, and gene order, the core block including *tadZ*, *tadA*, *tadB*, and *tadC* was consistently conserved.

Immunization with recombinant TadZ resulted in a significant reduction in mortality among challenged fish, yielding a relative percent survival (RPS) of 67.2% compared to the NI group. This level of protection closely aligns with earlier research demonstrating the strong efficacy of recombinant antigens against *A. hydrophila* in catfish. For example, Abdelhamed et al. (2016 and 2019) demonstrated strong vaccine efficacy against the virulent *A. hydrophila* strain vAh ML09-119, reporting RPS values ranging from 59.8% to 95.4% with recombinant fimbrial proteins and achieving 89.2% RPS using a recombinant ATPase protein. These results further support the promise of TadZ and related recombinant antigens for significantly protecting catfish against MAS in aquaculture (3, 40). In a recent study, autogenous bivalent vaccine containing *Aeromonas* VH31 and VH 74 antigens, derived from 2 different strains, provided 100% relative percent survival (RPS) in striped catfish, while each monovalent vaccine achieved 62.5% protection (41). When comparing vaccine efficacy across different studies, it is important to recognize that multiple experimental variables may influence outcomes. Factors such as sample size, fish species, bacterial strain, timing between vaccination and challenge, challenge dose, route of administration, the presence or absence of booster immunization, and the specific antigen used can all impact observed vaccine efficacy. In the present study, an RPS of 67.2% was achieved using a single immunization with recombinant TadZ protein, without a booster. This level of protection falls within the range previously documented in similar studies, highlighting the importance of considering methodological differences when interpreting and comparing vaccine performance. Various vaccine strategies against *A. hydrophila*, including recombinant outer membrane and ATPase proteins (3, 40, 42) and autogenous formulations derived from field isolates (41), have demonstrated protective efficacy in experimental catfish models. Broader considerations regarding vaccine platform selection, including safety, regulatory, and production aspects, have been discussed in recent reviews (12, 43) (12, 44). In this context, TadZ represents a conserved and functionally important antigen that warrants further evaluation within recombinant immunization approaches.

In addition, TadZ immunization reduced bacterial concentrations in the liver, anterior kidney, and spleen following challenge with vAh ML09-119, with a significant reduction observed in the liver and downward trends in the kidney and spleen. These findings are consistent with previous reports of vaccine-reduced bacterial concentrations in critical organs, including reductions in catfish vaccinated with recombinant outer membrane proteins (42) and a recombinant ATPase subunit (3). Bacterial load is a critical parameter for evaluating fish vaccine efficacy because it directly reflects the ability of the host immune system, stimulated by vaccination, to control and clear the infecting pathogen. A significant reduction in bacterial load in vaccinated fish indicates effective immune-mediated inhibition of pathogen proliferation within vital organs. This reduction supports the observed RPS results by demonstrating that vaccinated fish not only survive better but also harbor fewer bacteria, lowering disease severity and transmission risk.

In our study, the significant bacterial load reduction in the liver, coupled with downward trends in the kidney and spleen, provides biological evidence supporting the protective potential of the TadZ antigen. Because the liver is a primary immune organ responsible for filtering blood and clearing pathogens, enhanced bacterial clearance here is likely to play a pivotal role in reducing systemic infection and mortality (45, 46). Although reductions in kidney and spleen bacterial load were less pronounced, they may become significant with larger sample sizes or extended monitoring, potentially reflecting progressive pathogen elimination. Thus, bacterial concentration measurements complement survival data by revealing how effectively the TadZ antigen-induced immune response limits pathogen dissemination and growth in critical tissues. Together, these metrics provide a more comprehensive evaluation of antigen performance and inform strategies for optimizing its development as a vaccine candidate for durable protection. Although culture-based quantification was used to measure viable bacterial burden in this acute challenge model, future studies incorporating qPCR-based detection of vAh-specific genes may provide enhanced sensitivity for assessing low-level or persistent infection dynamics.

In the present study, antibody responses were assessed using a fixed serum dilution ELISA to enable consistent comparison among treatment groups under identical assay conditions. This approach allowed reliable detection of relative differences in antigen-specific IgM responses within this efficacy-focused screening framework. The strong IgM responses elicited by TadZ vaccination, observed both after immunization and following challenge, underscore its robust immunogenicity despite its intracellular localization. Similar findings have been reported for other conserved intracellular proteins, such as the cytosolic chaperone DnaK (Hsp 70) in fish vaccines (47, 48). These parallels reinforce the idea that TadZ, although cytoplasmic, can function as an effective vaccine antigen. After wild-type vAh strain ML09–119 challenge, IgM binding to the WT whole-cell antigen increased in both non-immunized controls and TadZ immunized fish, however, titers in the non-immunized group remained lower than in TadZ-immunized fish, consistent with vaccine priming that enabled a more rapid and robust humoral reaction upon challenge. The recognition of TadZ-specific antibodies despite its cytoplasmic localization is likely due to bacterial lysis, which exposes internal antigens to the immune system, thereby facilitating antibody recognition. In addition to its immunogenicity, TadZ’s biological function reinforces its potential as a vaccine target. Mutations in tad operon genes, including tadZ, reduce virulence and impair biofilm formation (19, 26, 35), suggesting that immune targeting of TadZ could weaken colonization and bacterial fitness. Antibody titers were slightly lower two days post-challenge than at three weeks post-immunization, reflecting the early phase of a secondary response, which typically requires several days to peak in fish (44, 49). Nevertheless, the presence of TadZ-specific antibodies at this early stage demonstrates effective immune priming, an important advantage given *A. hydrophila’s* ability to rapidly suppress host immunity (43, 50).

In summary, this study demonstrates that recombinant TadZ functions as a protective and immunogenic component of the Tad pilus system in catfish, inducing strong antigen-specific humoral responses, reducing mortality, and lowering bacterial burdens following challenge with virulent *A. hydrophila*. Although immune mechanisms remain to be fully defined, the protection observed may reflect a combination of humoral and innate responses together with interference in pilus-mediated virulence. Despite limitations such as pooled serum analysis, a single vaccine dose, and early sampling time points, these results highlight TadZ’s evolutionary conservation, critical role in pilus biogenesis, and immunogenic potential.

The present study was designed as an efficacy-focused screening study with survival, early post-challenge organ bacterial burden, and antigen-specific IgM responses as primary endpoints. Comprehensive clinicopathological characterization was not included in this study and represents an important area for future investigation to better define disease progression and TadZ-associated protective mechanisms.

Future studies should also focus on optimizing formulation and delivery strategies, including evaluation of adjuvants, booster schedules, and oral or immersion routes, to improve protection and field applicability. Comparative analyses with other Tad-associated proteins may identify conserved antigenic targets and guide the development of multivalent or cross-protective approaches. Overall, TadZ represents a promising antigen for broad vaccine applications in aquaculture. Collectively, our findings support TadZ as a promising immunogenic antigen for aquaculture with potential broader applicability to other pathogens that utilize Tad-associated systems.

## Data Availability

The datasets analyzed for this study can be found in the NCBI database. The accession numbers are as follows: NC021290.1; NC009348.1; NC015424.1; NC004459.3; NC004460.2; CP000020.2; CP000021.2; NZCP009538.1; NZCP009537.1; NZCP007502.1; NC002940.2; NC002663.1; NC015224.1; CP000720.1; AE005673.1; NZCP023315.3; NZCP116810.1; NZCP007744.1; AE004091.2; NZCP031787.1; BX470249.1; NC017218.1.
