# Supplementary material for: Recombinant TadZ from the type IVc pilus system induces protective immunity against virulent Aeromonas hydrophila in channel catfish (Ictalurus punctatus)
Source: Front Immunol. 2026 May 7;17:1763508. doi: 10.3389/fimmu.2026.1763508 (PMC13189830; doi:10.3389/fimmu.2026.1763508)
Supplement: Supplementary file 1 [file Table1.docx]

**Supplementary Material**

Table S1. Genomic features and Tad locus sizes of bacterial species analyzed in this study.

| No. | Bacterial species carrying Tad operon | Accession No. | Tad locus size (bp) | Genome size (bp) |
| --- | --- | --- | --- | --- |
| 1 | *Aeromonas hydrophila* ML09-119 | [NC_021290](https://www.ncbi.nlm.nih.gov/nuccore/NC_021290.1) | 10,853 | 5,024,500 |
| 2 | *Aeromonas salmonicida* subsp. salmonicida A449 | [NC_009348](https://www.ncbi.nlm.nih.gov/nuccore/NC_009348.1) | 9,467 | 4,702,402 |
| 3 | *Aeromonas veronii* B565 | [NC_015424](https://www.ncbi.nlm.nih.gov/nuccore/NC_015424.1) | 9,608 | 4,551,783 |
| 4 | *Vibrio vulnificus* CMCP6 chromosome I, 1^st^ Tad operon | [NC_004459](https://www.ncbi.nlm.nih.gov/nuccore/NC_004459.3) | 11,628 | 3,281,866 |
| 5 | *Vibrio vulnificus* CMCP6 chromosome I, 2^nd^ Tad operon |  | 9,322 |  |
| 6 | *Vibrio vulnificus* CMCP6 chromosome II | [NC_004460](https://www.ncbi.nlm.nih.gov/nuccore/NC_004460.2) | 11,327 | 1,844,830 |
| 7 | *Vibrio fischeri* ES114 chromosome I | [CP000020](https://www.ncbi.nlm.nih.gov/nuccore/CP000020.2) | 10,783 | 2,897,536 |
| 8 | *Vibrio fischeri* ES114 chromosome II | [CP000021](https://www.ncbi.nlm.nih.gov/nuccore/CP000021.2) | 10,654 | 1,330,333 |
| 9 | *Burkholderia pseudomallei* K96243 chromosome I | [NZ_CP009538](https://www.ncbi.nlm.nih.gov/nuccore/NZ_CP009538.1) | 11,492 | 4,074,547 |
| 10 | *Burkholderia pseudomallei* K96243 chromosome II | [NZ_CP009537](https://www.ncbi.nlm.nih.gov/nuccore/NZ_CP009537.1) | 13,256 | 3,173,067 |
| 11 | *Aggregatibacter actinomycetemcomitans* HK1651 | [NZ_CP007502](https://www.ncbi.nlm.nih.gov/nuccore/NZ_CP007502.1) | 11,516 | 2,105,503 |
| 12 | *Haemophilus ducreyi* 35000HP | [NC_002940](https://www.ncbi.nlm.nih.gov/nuccore/NC_002940.2) | 11,767 | 1,698,955 |
| 13 | *Pasteurella multocida* subsp. *multocida* str. Pm70 | [NC_002663](https://www.ncbi.nlm.nih.gov/nuccore/NC_002663.1) | 11,644 | 2,257,487 |
| 14 | *Yersinia enterocolitica* subsp. *palearctica* 105.5R(r) | [NC_015224](https://www.ncbi.nlm.nih.gov/nuccore/NC_015224.1) | 10,508 | 4,552,107 |
| 15 | *Yersinia pseudotuberculosis* IP 31758 | [CP000720](https://www.ncbi.nlm.nih.gov/nuccore/CP000720.1) | 9,078 | 4,723,306 |
| 16 | *Caulobacter crescentus* CB15 | [AE005673](https://www.ncbi.nlm.nih.gov/nuccore/AE005673.1) | 12,634 | 4,016,947 |
| 17 | *Caulobacter vibrioides* CB13b1a | [NZ_CP023315](https://www.ncbi.nlm.nih.gov/nuccore/NZ_CP023315.3) | 12,878 | 4,144,051 |
| 18 | *Rhodopseudomonas palustris* CGA009 | [NZ_CP116810](https://www.ncbi.nlm.nih.gov/nuccore/NZ_CP116810.1) | 11,521 | 5,459,214 |
| 19 | *Pectobacterium atrosepticum* JG10-08 | [NZ_CP007744](https://www.ncbi.nlm.nih.gov/nuccore/NZ_CP007744.1) | 12,586 | 5,004,926 |
| 20 | *Pseudomonas aeruginosa* PAO1 | [AE004091](https://www.ncbi.nlm.nih.gov/nuccore/AE004091.2) | 9,798 | 6,264,404 |
| 21 | *Bordetella pertussis* Tohama | [NZ_CP031787](https://www.ncbi.nlm.nih.gov/nuccore/NZ_CP031787.1) | 12,125 | 4,102,412 |
| 22 | *Bordetella parapertussis* 12822 | [BX470249](https://www.ncbi.nlm.nih.gov/nuccore/BX470249.1) | 12,155 | 4,773,551 |
| 23 | *Bifidobacterium breve* ACS-071-V-Sch8b | [NC_017218](https://www.ncbi.nlm.nih.gov/nuccore/NC_017218.1) | 4,641 | 2,327,492 |
